# Involvement in emergency situations by primary care doctors on-call in Norway - a prospective population-based observational study

**DOI:** 10.1186/1471-227X-10-5

**Published:** 2010-03-06

**Authors:** Erik Zakariassen, Steinar Hunskaar

**Affiliations:** 1 Department of Research, Norwegian Air Ambulance Foundation, Box 94, Drøbak, Norway; 2 National Centre for Emergency Primary Health Care, Kalfarveien 31, 5018 Bergen, Norway; 3 Section for General Practice, Department of Public Health and Primary Health Care University of Bergen, Kalfarveien 31, 5018 Bergen, Norway

## Abstract

**Background:**

Primary care doctors on-call in the emergency primary health care services in Norway are, together with the ambulances, the primary resources for handling emergencies outside hospitals. There is a lack of reliable data for Norway on how often the primary care doctors are alerted and on their responses in the most urgent emergency cases. The aim of this study was to investigate how doctors on-call are involved in red responses (highest priority), using three different emergency medical communication centres (EMCC) as catchment area for a prospective population-based study.

**Methods:**

In the period from October to December 2007 three dispatch centres covering approximately 816 000 inhabitants prospectively recorded all acute emergency cases. Ambulance records, air ambulance records and records from the doctors on-call were collected. NACA score was used to define the severity of the emergencies.

**Results:**

5 105 cases were classified as red responses during the period. We have complete basic recordings (AMIS forms) from all and resaved ambulance records, air ambulance records and records from doctors on-call in 89% of the cases. Ambulances were alerted in 96% and doctors on-call in 47% of the cases, but there were large differences between the three EMCCs. Doctors on-call responded with call-out in 42% of the alerted cases. 28% of all patients were taken to a casualty clinic, 46% were admitted to hospital by a doctor and 24% were taken directly to hospital by ambulances. In total, primary care doctors on-call took active part in 42% of all red response cases, and together with GPs' daytime activity the primary health care services were involved in 50% of the cases. 29% of the cases were classified as life-threatening. Call-out by doctors on-call were found to be more frequent in life-threatening situations compared with not life-threatening situations.

**Conclusion:**

Doctors on-call and GPs on daytime were involved in half of all red responses. There were large differences between the EMCCs in the frequency of doctors alerted. The inhabitants in the three EMMCs were thus offered different levels of professional competency in emergency situations outside hospitals.

## Background

The primary resources in the Norwegian pre-hospital emergency care system are the ambulances and the primary care doctors. Ambulance personnel and primary care doctors on-call thus constitute a major part in the "chain of survival", the doctors being especially present as an important resource in rural areas [1].

In Norway, the municipalities are responsible for the emergency primary healthcare system, including the out-of-hours services, primary care doctors on-call, casualty clinics and local emergency medical communication centres (LEMC) [2]. The doctors have an obligation to take part in the restricted and nationwide medical radio network (radio) used as the national standard for communication between doctors on-call, ambulance personnel and the emergency medical communication centres (EMCC) (dispatch centrals) [3].

The central government is responsible for the secondary health care system; hospitals, EMCCs, ground and boat ambulances and the national air ambulance service, staffed with anaesthetists. An important principle in the health care system in Norway is the gatekeeper function exerted by the primary care doctors; patients cannot meet directly at hospitals without being referred by a doctor. However, in a severe emergency situation the ambulance may drive directly to hospital without a doctor's confirmation, but then only in agreement with health personnel in the EMCCs.

A national three digits emergency number (113) to an EMCC is used when medical emergencies occur. All EMCCs use a software system called Acute Medical Information System (AMIS) to record the cases, and they use the Norwegian Index of Medical Emergencies (Index) [4] as a decision tool for level of emergency. Based on the Index the EMCC nurses will classify the call as a "red response", with highest priority; "yellow response", urgent but not acute; or "green response", with lowest priority. If Index prescribes a red response, a radio alarm alert shall be sent simultaneously to the doctor on-call and the ambulances in the actual geographical area.

Ambulance personnel have argued that primary care doctors on-call leave the responsibility of the emergency patients more frequently to them, compared to earlier [1]. Only half of the out-of-hours districts in Norway had doctors who always used the radio in 2005 [5]. A study found geographical differences in the involvement of Norwegian doctors on-call in pre-hospital emergencies, but the study was limited to situations where the air ambulances were alerted as well [6]. Two studies have investigated rGPs'experiences with emergency situations, though not red responses in particular, through the EMCC system [7,8]. On a national basis the EMCCs in Norway alerted doctors on-call in about 50% of the red response cases [9]. A recent study describes difficulties in cooperation between doctors on-call and ambulance personnel [10].

The aim of this study was to investigate how red response situations are administrated with special focus on the primary care doctors on-call, using three different EMCCs as catchment area for a prospective population-based study.

## Methods

For data collection we chose the EMCCs at Haugesund, Stavanger and Innlandet hospitals. Together they cover 69 581 km^2 ^(21% of total area of Norway), 816 000 inhabitants (18% of total) and 85 municipalities (20% of total). The out-of-hours districts, 34 in total, are both single-municipal and inter-municipal, rural and city areas.

To secure a uniform use of the AMIS program a meeting between the leaders of the EMCCs was arranged. The AMIS forms contained information on date, time of day, time for alerts to the different pre-hospital recourses, who responded, response time, criteria code for the emergency cases and to where the patients were transported. AMIS forms and copies of ambulance records from all red responses were submitted to the project manager every second week. In the cases where doctors on-call or air ambulances had been involved, copies of medical records were requested by mail. Data registration period lasted from October 1^st ^to December 31^st ^2007. Collection of medical records from different parts of the health care system was made until October 2008.

From the retrieved records we extracted the information needed to classify the severity of the medical problems based on The National Committee on Aeronautics Score System (NACA) [11]. NACA score were in the analyses dichotomised into not life-threatening (NACA value 0-3) and life-threatening or dead (NACA value 4-7). Data on municipalities were obtained from Statistics Norway. Municipal centrality is categorised with values from zero to three. This variable was then dichotomised into remote (value 0-1) and central municipalities (value 2-3).

The statistical analyses were performed using Statistical Package for the Social Sciences (SPSS version 15). Standard univariate statistics were used to characterise the sample. Data are presented as mean (SD). Skewed distributed data are presented as median with 25-75% percentiles. Differences between variables were analysed using Pearson's χ^2 ^test. Fisher's exact test was computed when tables had cells with a frequency of less than five in 2 × 2 tables. P value < 0.05 was considered as statistically significant. Rate is presented as numbers of red responses per 1 000 inhabitants per three months. Logistic regression analyses were used to calculate the odds ratio (OR) for alerts sent to doctors on-call and doctors' responses to the alerts. Cases without an alert sent to a doctor are excluded from the regression analyses together with secondary air ambulance missions (transfer between hospitals). The dependent variable "doctor's response" was dichotomised into "call-out" or not, "await" or not and "consult" or not. Air ambulance on call-out (yes or no), the dichotomised versions of NACA score, municipal centrality (dichotomised), and the variable "populations in the primary care district" were used as independent variables in the analyses. "Populations in the primary care district" was divided into five categories, with value 1 including 0-25 000, value 2 including 25 001-50 000, value 3 including 50 001-75 000, value 4 including 75 001-100 000 and value 5 >100 000 inhabitant. Cases where a doctor was the caller to the EMCC are left out in some of the analyses, because there is no need to alert the doctor when the doctor already knows about the situation.

Approval of the study was given by the Privacy Ombudsman for Research, Regional Committees for Medical Research Ethics and Norwegian Directorate of Health.

## Results

During the three months of inclusion 5 105 red responses with AMIS forms were recorded and included. In 4 551 (89%) of the forms we retrieved one or more extra records belonging to same case. Total rate (per 3 months) of red responses was 6.2 per 1 000 inhabitants.

Next of kin was the main caller to the EMCCs. Health care personnel, LEMCs and doctors made more than a third of the calls for ambulances (table 1). Ambulances were alerted in nearly all the red response cases and doctors on-call in nearly half of the cases. Doctors on-call responded with call-out in 42% of the cases in which they were alerted. Differences between the EMCC districts are pronounced with respect to alerting doctors on-call. EMCC Innlandet alerted doctors on-call in a fifth of the cases compared with three out of four of the cases in Stavanger and Haugesund, but there were no statistical significant differences in call-out as response when an alert was given (p = 0.056).

**Table 1:** Red responses distributed by caller, alert and responses

	Total		Innlandet		Stavanger		Haugesund	
	
	n	%	n	%	n	%	n	%
**Caller to the EMCCs**								
Next of kin	1 705	34	899	34	520	35	286	31
Bystander	857	17	411	16	263	18	183	20
Health personnel	914	18	523	20	229	15	162	17
Doctor	455	9	267	10	95	6	93	10
LMCC	451	9	206	8	192	12	53	6
Patient	349	7	144	6	117	8	88	10
Police	221	4	115	4	57	4	49	5
Fire department	87	2	58	2	23	2	6	1
**Alerted**								
Ambulance	4 896	96	2 549	97	1 457	95	890	96
GP on call*	2 105	47	469	21	1 047	75	589	72
Air ambulance#	377	8	118	5	201	15	58	7
Anaesthetist (from hospital)	92	2	89	3	0	0	0	0
Fire department	210	4	122	5	47	3	32	3
Police	314	6	160	6	89	6	65	7
**Doctors' response**†								
Call out	829	42	176	47	434	41	219	40
Await	750	37	96	26	471	44	183	33
Confer	287	14	88	24	62	6	137	25
No contact	94	5	11	3	69	6	14	2
Occupied	35	2	3	~0	30	3	2	~0
Total¤	1 995	100	374	100	1 066	100	555	100

¤Differences in numbers between alerted doctors and total numbers on responses are due to missing data in both variables

*Selected cases without doctors as caller

#Selected cases without secondary missions (transfer between hospitals)

† Selected cases where GPs were alerted

In 9% of the cases a doctors was the caller to the EMCC (table 1). Other health care personnel and LEMCs called for ambulances in 27% of the cases, and thus patients, next of kin and bystanders were the callers in less than 60% of the incidents. More than half (55%) of the calls from doctors to the EMCCs were during daytime, 33% in the evenings and 12% during the night. Patient's location when doctors were callers was in 42% of the cases private homes, 9% casualty clinics, 22% doctors' surgeries, 20% hospitals and nursing homes, and other locations in 7% of the cases. When the EMCCs alerted the doctors the distribution of alerts was 37% for both daytime and evenings, and 26% during nights. When doctors on-call were alerted, the location of the patient was a private home in 63% of the cases, 30% was public places, 4% nursing homes, and 3% other places. Doctors on-call were alerted median 0 minutes (0-2) after the ambulances, 57% at the same time and 86% within the first five minutes. Innlandet alerted 67% during the first 5 minutes after the ambulances had been alerted, Stavanger 95% and Haugesund 83% (p < 0.001). Doctors on-call were alerted after the arrival of ambulances to patients in 3% of the cases.

In cases when the air ambulance/anaesthetist was alerted (transfer between hospitals are excluded) the EMCCs also alerted doctors on-call in 72% of the cases and doctors on-call responded with a call-out in 62% of those cases. Innlandet alerted doctors on-call in 38% of the same cases as the air ambulances/anaesthetist, Haugesund 68% and Stavanger 78% (p < 0.000). The doctors on-call responded in 64% of the same cases as the air ambulance/anaesthetist in Innlandet, 72% in Haugesund and 53% in Stavanger (p < 0.04).

Primary care doctors' involvement in the treatment and the decision regarding the location to which the patients were transported are shown in table 2. In situations where doctors on-call were not alerted patients were transported directly to hospitals with ambulance twice as often compared to situations where doctors were alerted. 26% of all patients were transported to casualty clinics independently of whether the doctors on-call were alerted or not. When doctors responded with call-out, more than half of the patients were admitted to hospitals, and when "await" was the response more than 43% of the patients were taken to casualty clinics. When doctors called the EMCCs the majority of the patients were admitted to hospital by doctor's referral. In both the not life-threatening and the life-threatening cases a fourth of the patients was transported with ambulances directly to hospitals without any involvement of doctors. Doctors on-call were involved in 42% of all red response cases. Including daytime activity among rGPs the primary health care services were involved in 50% of the cases.

**Table 2:** Involvement of doctors and locations for transport of patients

Patients transported to	Casualty clinic		Hospital via casualty clinic		Admitted to hospital by doctors		Directly to hospital by ambulance		Patients stayed at scene		Dead patients		Follow up by others		Total*	
	**n**	**%**	**n**	**%**	**n**	**%**	**n**	**%**	**n**	**%**	**n**	**%**	**n**	**%**	**n**	**%**
	
Doctors were callers	22	5	30	7	356	80	16	4	20	4	3	~0	0	0	447	100
Doctors alerted§																
Yes	328	15	270	13	781	37	334	16	283	13	117	5	23	1	2 136	100
No	403	14	250	9	934	34	837	31	239	9	50	2	18	1	2 731	100
Total	731	15	520	11	1 715	35	1 171	24	522	11	167	3	41	1	4 867	100
Doctors' response when alerted																
Call out	75	9	41	5	455	56	7	1	128	16	98	12	6	1	810	100
Await	157	21	162	22	142	19	205	28	55	8	10	1	9	1	740	100
Consult	32	11	25	9	128	45	23	8	72	25	2	1	2	1	284	100
No contact	20	22	11	12	16	18	30	33	12	13	1	1	1	1	91	100
Occupied	8	23	11	31	7	20	8	23	1	3	0	0	0	0	35	100
Total*	292	15	250	13	748	38	273	14	268	14	111	5	18	1	1 960	100

* Differences in numbers between alerted doctors and doctors response are due to missing data

§Doctors as callers are excluded

The frequency of alert and responses from the doctors on-call by central and remote municipalities are shown in table 3. Alert to doctors on-call was highest in central municipalities in all EMCC areas, although not statistically significant in Stavanger area. However, the number of responses with call-out is higher in remote compared to central municipalities, with smallest difference appearing in Haugesund.

**Table 3:** Alerts and responses by rural and central municipalities

				Response if alerted^§^											
	
	Doctors alerted*			Call-out			Await			Consulted			Other^β^		
**Municipal centrality**	**n**	**%**	**P-** **value**	**n**	**%**	**P-** **value**	**n**	**%**	**P-** **value**	**n**	**%**	**P-** **value**	**n**	**%**	**P-** **value**
	
Innlandet (n = 461)															
Remote	387	19		147	49		70	23		72	24		13	4	
Central	74	28	0.00	19	32	0.02	25	42	0.00	15	25	0.74	1	1	0.36
Stavanger (n = 1058)															
Remote	71	70		39	57		12	18		10	15		7	10	
Central	987	76	0.22	381	40	0.00	439	46	0.00	50	5	0.00	89	9	0.81
Haugesund (n = 586)															
Remote	529	68		193	40		168	35		109	22		16	3	
Central	57	84	0.01	20	36	0.63	12	22	0.10	23	42	0.00	0	0	0.18
Total															
Remote	987	33		379	45		250	29		191	22		36	4	
Central	1 118	68	0.00	420	39	0.02	476	45	0.00	88	8	0.00	90	8	0.00

*Doctors as callers were excluded from analyses

§Differences between numbers for alerted doctors and total numbers for responses are due to missing data

β"Occupied" and "No contact" were merged to "Other"

The distribution of doctors as caller, alerted doctors and doctors' response between life and not life-threatening situations is shown in table 4. When doctors were the callers the majority of the cases were not life-threatening situations. Stavanger EMCC had the highest percentage of alerted doctors in both life-threatening and not life-threatening situations. Innlandet EMCC had the largest difference in alerts between life and not life-threatening conditions. Overall, differences in call-outs between life-threatening and not life-threatening conditions are pronounced when doctors are alerted. In not life-threatening conditions the response "await" was most frequent. In life-threatening conditions doctors on-call in Innlandet responded considerably more often with call-outs when compared to Stavanger and Haugesund. Doctors in the Stavanger area had the highest percentage of "await" as response.

**Table 4:** Alerts and responses for not life-threatening (NACA 0-3) and life-threatening situations (including death) (NACA 4-7)

			**Innlandet**				**Stavanger**				**Haugesund**			
	
	**Total**		**Not life-threatening**		**Life-threatening**		**Not life-threatening**		**Life-threatening**		**Not life-threatening**		**Life-threatening**	
	
	**n**	**%**	**n**	**%**	**n**	**%**	**n**	**%**	**n**	**%**	**n**	**%**	**n**	**%**
	
Doctors were callers	403	100	152	61	96	39	48	65	26	35	51	63	30	37
Doctors on call alerted^§^														
Yes	1 881	47	277	18	146	26	676	77	255	78	366	73	161	69
No	2 162	53	1 271	82	411	74	200	23	71	22	135	27	73	31
Total	4 042	100	1 548	100	557	100	876	100	326	100	501	100	234	100
Doctors' response when alerted														
Call out	778	43	85	38	81	64	265	40	143	56	122	36	87	54
Await	639	36	71	32	20	16	321	47	83	32	126	37	36	22
Consult	287	16	57	25	26	20	39	6	12	5	83	24	34	22
No contact	70	4	9	4	0	0	37	5	14	5	10	3	2	1
Occupied	26	1	2	1	0	0	17	2	5	2	1	~0	1	1
Total*	1 800	100	224	100	127	100	679	100	257	100	342	100	160	100

*Differences in numbers between alerted doctors and total numbers on responses are due to missing data

§Doctors as caller are excluded

Overall, 70% of all alerts sent to doctors on-call were for not life-threatening conditions, and 61% of all call-outs among the doctors on-call occurred in not life-threatening situations. In total, EMCCs alerted doctors on-call in half of the life-threatening situations, compared to 45% in not life-threatening situations (p < 0.004). Doctors on-call responded with call-outs in 56% of the life-threatening situations compared to 38% in not life-threatening situations (p < 0.000).

By regression analyses clear associations were found between EMCC areas and whether the doctors on-call were alerted or not. There is also a statistical significant association between alerts in not life-threatening situations and alerts to primary care doctors in remote municipalities (table 5).

**Table 5:** Odds ratio (95% CI) for primary care doctors being alerted

	**Doctors alerted †**
	
Dispatch centrals and area	
Haugesund	1
Stavanger	8.58 (6.98-10.6)
Innlandet	0.91 (0.66-1.24)
Not life-threatening condition (NACA) ¤	1.28 (1.08-1.52)
Remote municipalities ¤	2.59 (2.00-3.35)
No use of radio among doctors on-call ¤	0.76 (0.62-0.95)
Population in the primary care districts	1.30 (1.22-1.39)

† Selected cases; Doctors as caller to the EMCCs are excluded

¤ Dichotomised variables, reference value = 1

Low severity score on NACA were associated with a higher possibility of call-out as response among the primary care doctors. There was a positive statistically significant association between call-out and remote municipalities in the total area, but when each district was analysed this association was significant only for Stavanger. For the total area the air ambulance on call-out was associated with a statistically significant decrease in odds ratio for primary care doctors being on call-out to the same patients, but the results were not statistically significant for the Stavanger area. Increasing population in the primary care districts is associated with more call-outs as the response among the primary care doctors in all three areas (table 6).

**Table 6:** Odds ratios for (95% CI) type of response when primary care doctors were alerted for total area and in the three EMCC districts

Doctors responses*	Call-out	Await	Confer
Total area			
Not life-threatening condition (NACA) ¤	2.11 (1.69-2.62)	0.53 (0.42-0.67)	1.02 (0.76-1.39)
Air ambulances on call-out ¤	0.64 (0.46-0.89)	1.22 (0.86-1.74)	4.02 (1.93-8.41)
Population in the primary care districts	1.41 (1.30-1.53)	0.74 (0.75-1.31)	1.01 (0.90-1.13)
Remote municipalities ¤	2.10 (1.58-2.79)	0.99 (0.68-0.80)	0.36 (0.24-0.53)
Area of Innlandet			
Not life-threatening condition (NACA) ¤	2.62 (1.60-4.29)	0.45 (0.25-0.80)	0.97 (0.57-1.66)
Air ambulances on call-out ¤	0.31 (0.13-0.73)	1.36 (0.54-3.44)	10.6 (1.42-78.9)
Population in the primary care districts	1.21 (1.01-1.46)	1.02 (0.84-1.25)	0.83 (0.68-1.00)
Remote municipalities ¤	0.66 (0.32-1.35)	2.35 (1.21-4.54)	0.81 (0.40-1.61)
Area of Stavanger			
Not life-threatening condition (NACA) ¤	1.80 (1.30-2.47)	0.63 (0.45-0.87)	0.86 (0.43-1.70)
Air ambulances on call-out¤	0.94 (0.60-1.46)	1.17 (0.75-1.85)	2.52 (0.74-8.62)
Population in the primary care districts	1.71 (1.49-1.95)	0.58 (0.50-0.69)	1.06 (0.85-1.33)
Remote municipalities¤	3.70 (1.79-7.67)	0.51 (0.21-1.25)	0.37 (0.13-1.08)
Area of Haugesund			
Not life-threatening condition (NACA) ¤	1.99 (1.32-3.00)	0.58 (0.37-0.90)	0.99 (0.61-1.60)
Air ambulances on call-out¤	0.45 (0.21-0.98)	1.73 (0.68-4.40)	2.08 (0.69-6.30)
Population in the primary care districts	1.30 (1.07-1.57)	0.94 (0.78-1.14)	0.76 (0.61-0.94)
Remote municipalities¤	0.97 (0.52-1.82)	0.53 (0.26-1.09)	2.45 (1.31-4.56)

* Selected cases; doctors not alerted in the primary care system are excluded

¤ Dichotomised variables, reference value = 1

## Discussion

Primary care doctors in the health care services, including rGPs during daytime and primary care doctors on-call out-of-hours, took active part in 50% of all red responses. Primary care doctors on-call were alerted in nearly half of the red response cases managed by the three EMCCs. The doctors on-call responded with call-outs or consulted the ambulance personnel in a majority of the alerted cases, and they responded with call-outs in more than 55% of the life-threatening situations in all three areas. There were significant differences in the proportion of alerted doctors between the EMCCs. If alerted, however, the response pattern was similar.

The strengths of our study include its completeness, representativity, and number of variables included. In the course of a three month period we were able to prospectively collect a complete material of more than 5 000 red responses based on a population of 816 000 inhabitants, close to 20% of the Norwegian population. The three EMCCs and their actions may not be representative for all EMCCs in Norway. Taken together, however, the three EMCCs have the same frequency of alerted doctors as was found in a national survey [9]. The minor differences between the three EMCC areas with respect to doctors' responses strengthen the representativeness of the 85 municipalities and 35 out-of-hours districts. In nearly 90% of all cases we retrieved records from car and air ambulances, casualty clinics and rGPs. Together with the complete set of AMIS forms this yields a comprehensive material for analysis of the objectives of the study. Severity score (NACA) on patients was assessed retrospectively based on medical records and may therefore have lower accuracy. It is also a limitation of the study that we lack access to the patients' medical records after hospitalisation. Analyse of the medical usefulness of having the primary care doctor at site was thus not possible.

The pronounced differences between the EMCCs with respect to alerting primary care doctors on-call indicate that the opportunity to have a doctor on scene as part of the initial examination and treatment varies among the inhabitants in different geographical areas. The government wants to have a decentralised pattern of settlement in Norway and obtaining equality in health care is a stated political goal [12]. By not alerting the doctors on-call an EMCC violates the regulation for pre-hospital emergency [3] and the inhabitants are not offered an equal level of medical competency. The large majority of the patients are not in need of immediate treatment based on protocols, like cardiopulmonary resuscitation, and most patients are elderly with more complex medical symptoms and comorbidities [13]. Ambulance personnel's formal education is two years in upper secondary school and two years in apprenticeship [3]. However, a large group of ambulance personnel does not fulfil that educational level [14]. As patients in most cases of the emergency situations have complex medical problems [14,15] there is need of competence based on higher education and experience when examining the patients. Compared to ambulance personnel the doctors are superior when it comes to clinical judgement and deciding treatment and level of care when the patients have a serious illness. The professions provide supplementary contributions [15] and the professions should more frequently appear together on site. More doctors on site could possibly contribute to a reduction of transports to both casualty clinics and hospitals, thus decreasing hospitalisations and use of the ambulances. Direct transports to hospitals by the ambulance services were doubled when doctors on-call were not alerted, compared to whit if they were (table 2). This difference indicates the important gatekeeper function by the emergency primary healthcare services. Every patient treated by the primary care services will reduced costs because the patient is treated at a lower and less costly level of care. Still, differences in medical usefulness between patients transported directly to hospitals without a doctor's involvement and those admitted to hospitals by a doctor on-call is unknown. This should be addressed in further studies based on e.g. days spent in hospital before discharge.

For one third of all red response patients a professional medical judgement of the patient was made before the EMCCs were contacted. One third of the calls come from the primary health care system in the municipalities. Patients with serious illness can visit their rGP on daytime, and they may contact the casualty clinic or LEMC all hours. In addition home care nurses meet patients who are in need of immediate medical attention during their work. A study from Norway on incidences of emergency contacts (red responses) to the out-of-hours services found nearly the same volume of red responses as our study did [16].

The ambulance personnel transmit ECGs to hospitals and use doctors at the hospitals actively for consultations e.g. with regards to heart conditions and in order to decide what treatment to provide and where the patients should be transported [17]. This could be one reason for the small differences in the percentage of patients admitted to hospital by a doctor, regardless of whether the primary care doctors on-call were alerted or not.

When alerted, the doctors on-call in the remote areas responded more often with call-outs than doctors in more central municipalities. The regression analyses support the findings for the total catchment area, but there are differences between the three EMCC areas. The findings are similar to earlier studies [5-7]. Again, the levels of professional medical knowledge offered to the inhabitants vary due to different patterns of response among doctors on-call in different geographical areas.

Primary care doctors on-call were more often on call-out to patients with high NACA scores. This was most explicit in the EMCCs Innlandet. Innlandet had the lowest percentage of alerted doctors on-call, but the highest percentage of call-outs in life-threatening situations. Thus, there seems to be some pre-selection of the red response cases before doctors are alerted, which could give the doctors on-call an experience of higher accuracy on severity. In one remote municipality in Norway the doctors on-call defined 39% percent of all red response alerts as yellow (urgent, not acute) immediately after the situation was described via radio [12]. In our study 71% was classified as not life-threatening conditions and this could be one reason for "await" being the response in 37% of the cases. Other studies also describe overtriage in dispatch [18,19].

The association between specific EMCC districts and the probability of alerting doctors on-call is strong. The regression analyses also reflect that 71% of all red response cases were classified as not life-threatening. When doctors on-call were alerted and responded with call-out the large majority was done in not life-threatening situations. There is an association between alert and not life-threatening situations, and for the same reason the association between call-out and not life-threatening situations is strong in all three areas.

## Conclusions

Primary care doctors on-call and the primary health care system with rGPs on daytime took part in clinical judgement and treatment in half of all red response cases, and for one third of these a clinical judgement was made before an EMCC was contacted. The inhabitants in the catchment area were offered different levels of professional medical judgement and treatment. The EMCCs are not consistent with regards to alerting doctors on-call in red responses. There are differences between the EMCCs areas in terms of frequency of alerted primary care doctors on-call, but the type of response was more similar among the doctors.

## Competing interests

The authors declare that they have no competing interests.

## Authors' contributions

EZ and SH planned and established the project, including the procedures for data collection. EZ performed the analyses and drafted the manuscript. Both authors took part in the rewriting and approved the final manuscript.

## Pre-publication history

The pre-publication history for this paper can be accessed here:

http://www.biomedcentral.com/1471-227X/10/5/prepub
