# Correction: Involvement in emergency situations by primary care doctors on-call in Norway – a prospective population-based observational study

**DOI:** 10.1186/1471-227X-12-5

**Published:** 2012-05-08

**Authors:** Erik Zakariassen, Steinar Hunskaar

**Affiliations:** 1 Department of Research, Norwegian Air Ambulance Foundation, Box 94, Drøbak, Norway; 2 National Centre for Emergency Primary Health Care, Kalfarveien 31, 5018 Bergenr, Norway; 3 Section for General Practice, Department of Public Health and Primary Health Care University of Bergen, Kalfarveien 31, 5018 Bergen, Norway

## 

We have discovered that the regression analyses presented in Tables 1 and 2 in our original study [1] were not correct. The dependent variables were coded opposite of what intended. Below we present correct Tables 1 and 2. When comparing the original printed tables with the new ones, the reader will see that almost all odds ratios are inverted. For the interpretation of the results this means that some statements in the original paper should be changed to:

1. Alerting the primary care doctor was less likely if the event was not life-threatening and the primary care doctor was situated in a remote municipality.

2. Adjusted regression analyses showed that high severity score on NACA were associated with a higher possibility of call-out as the response among primary care doctors, but call-outs were less likely in remote municipalities.

3. Air ambulance mission was associated with a statistically significant increased risk of a call-out from the primary care doctor to the same patient.

4. Larger population in the primary care district was associated with less call-out as the response among the primary care doctors in all three areas.

**Table 1:** Odds ratio (95 % CI) for primary care doctors being alerted

	**Doctors alerted †**
Dispatch centrals and area	
Haugesund	1
Stavanger	1.10 (0.81-1.51)
Innlandet	0.12 (0.10-0.14)
Not life-threatening condition (NACA) ¤	0.78 (0.66-0.92)
Remote municipalities ¤	0.39 (0.30-0.50)
No use of radio among doctors on-call ¤	0.76 (0.61-0.94)
Population in the primary care districts	0.77 (0.72-0.82)

† Selected cases; Doctors as caller to the EMCCs are excluded.

¤ Dichotomised variables, reference value = 1.

**Table 2:** Odds ratios for (95 % CI) type of response when primary care doctors were alerted for total area and in the three EMCC districts

Doctors responses*	**Call-out**	**Await**	**Confer**
Total area			
Not life-threatening condition (NACA) ¤	0.51 (0.41-0.63)	1.78 (1.41-2.25)	1.02 (0.76-1.39)
Air ambulances on call-out ¤	1.53 (1.12-2.10)	0.78 (0.54-1.12)	4.02 (1.93-8.41)
Population in the primary care districts	0.74 (0.68-0.80)	1.34 (1.23-1.45)	1.01 (0.90-1.13)
Remote municipalities ¤	0.47 (0.36-0.62)	0.97 (0.73-1.29)	0.36 (0.24-0.53)
Area of Innlandet			
Not life-threatening condition (NACA)	0.39 (0.25-0.61)	2.23 (1.25-3.97)	1.03 (0.60-1.78)
Air ambulances on call-out ¤	2.16 (1.10-4.24)	0.73 (0.29-1.85)	0.10 (0.01-0.71)
Population in the primary care districts	0.82 (0.69-0.97)	0.98 (0.80-1.19)	1.21 (1.00-1.47)
Remote municipalities ¤	1.51 (0.78-2.93)	0.43 (0.22-0.83)	1.24 (0.62-2.48)
Area of Stavanger			
Not life-threatening condition (NACA)	0.58 (0.42-0.80)	1.60 (1.15-2.20)	1.17 (0.59-2.33)
Air ambulances on call-out¤	1.05 (0.68-1.62)	0.85 (0.54-1.34)	0.40 (0.12-1.36)
Population in the primary care districts	0.62 (0.55-0.70)	1.71 (1.45-2.01)	0.94 (0.75-1.18)
Remote municipalities¤	0.32 (0.16-0.62)	1.98 (0.80-4.88)	2.72 (0.92-8.03)
Area of Haugesund			
Not life-threatening condition (NACA)	0.46 (0.32-0.71)	1.74 (1.11-2.73)	1.02 (0.63-1.64)
Air ambulances on call-out¤	2.26 (1.01-4.81)	0.58 (0.23-1.48)	0.48 (0.16-1.46)
Population in the primary care districts	0.76 (0.64-0.92)	1.06 (0.88-1.29)	1.32 (1.07-1.64)
Remote municipalities¤	0.96 (0.52-1.79)	1.89 (0.91-3.90)	0.41 (0.22-0.76)

* Selected cases; doctors not alerted in the primary care system are excluded.

¤ Dichotomised variables, reference value = 1.

## Pre-publication history

The pre-publication history for this paper can be accessed here:

http://www.biomedcentral.com/1471-227X/12/5/prepub
